# The Cardioprotective Effect of Corosolic Acid in the Diabetic Rats: A Possible Mechanism of the PPAR-γ Pathway

**DOI:** 10.3390/molecules28030929

**Published:** 2023-01-17

**Authors:** Faisal K. Alkholifi, Sushma Devi, Hasan S. Yusufoglu, Aftab Alam

**Affiliations:** 1 Department of Pharmacology, College of Pharmacy, Prince Sattam Bin Abdulaziz University, Al Kharj 11942, Saudi Arabia; 2 Chitkara College of Pharmacy, Chitkara University, Rajpura 140401, Punjab, India; 3 Department of Pharmacognosy & Pharmaceutical Chemistry, College of Dentistry & Pharmacy, Buraydah Private Colleges, Buraydah 51418, Saudi Arabia; 4 Department of Pharmacognosy, College of Pharmacy, Prince Sattam Bin Abdulaziz University, Al-Kharj 11942, Saudi Arabia

**Keywords:** corosolic acid, streptozotocin, myocardial infarction, PPAR-γ, GW9662, isoproterenol

## Abstract

The study was conducted to determine whether corosolic acid could protect the myocardium of diabetic rats from damage caused by isoproterenol (ISO) and, if so, how peroxisome proliferator-activated receptor gamma (PPAR-γ) activation might contribute into this protection. Diabetes in the rats was induced by streptozotocin (STZ), and it was divided into four groups: the diabetic control group, diabetic rats treated with corosolic acid, diabetic rats treated with GW9662, and diabetic rats treated with corosolic acid plus GW9662. The study was carried out for 28 days. The diabetic control and ISO control groups showed a decrease in mean arterial pressure (MAP) and diastolic arterial pressure (DAP) and an increase in systolic arterial pressure (SAP). The rat myocardium was activated by corosolic acid treatment, which elevated PPAR-γ expression. A histopathological analysis showed a significant reduction in myocardial damage by reducing myonecrosis and edema. It was found that myocardial levels of CK-MB and LDH levels were significantly increased after treatment with corosolic acid. By decreasing lipid peroxidation and increasing endogenous antioxidant levels, corosolic acid therapy showed a significant improvement over the ISO diabetic group. In conclusion, our results prove that corosolic acid can ameliorate ISO-induced acute myocardial injury in rats. Based on these results, corosolic acid seems to be a viable new target for the treatment of cardiovascular diseases and other diseases of a similar nature.

## 1. Introduction

The most common causes of death and disability for diabetic patients are myocardial infarction (MI), angina, ischemic heart failure, and arrhythmias. Acute myocardial necrosis is caused by an imbalance between the demand of the heart muscle and the coronary blood flow [1,2]. The increased free radical formation, apoptosis, infiltration of inflammatory cells, and persistent DNA damage are part of a complex web of pathogenic pathways that contribute to the development of MI [3]. Diabetes contributes to higher postinfarction morbidity in individuals who have already suffered an acute MI and is associated with a higher mortality rate in these patients [4]. The development of diabetes and its associated cardiovascular complications can be linked to oxidative stress and increased apoptosis [5]. Chronic hyperglycemia and advanced glycation end products are responsible for the formation of superoxide anions and reactive oxygen species (ROS), both of which contribute to an increased risk of developing cardiovascular disease [6]. Therefore, people with diabetes have a higher risk of morbidity and a higher likelihood of developing MI. Diabetics had about twice the risk of death during hospitalization and follow-up for acute MI compared with nondiabetics [7].

The treatment and management of diabetic patients usually include using beta-blockers, angiotensin/angiotensin receptor conversion enzyme inhibitors (II), nitrates, and antithrombotic drugs. These drugs alleviate MI symptoms by increasing blood flow to the heart and protecting it from apoptotic damage [8,9]. Despite these treatment measures, the mortality rate for acute diabetes is still over 30% [10]. However, the effectiveness of existing drugs is very limited and has serious shortcomings. Therefore, a new drug/agent with minimal side effects is necessary to prevent and treat heart disease.

Natural products are considered promising for treating cardiovascular and related disorders in drug discovery. Most discovered compounds are selective peroxisome proliferator-activated receptor-gamma (PPAR-γ) modulators. Numerous compounds from medicinal plants and foods have explored the potential of PPAR-γ activation [11]. These compounds serve as partial agonists to transactivate the expression of reporter genes dependent on PPAR-γ. Some in-vivo results suggested that some natural products, PPAR-γ activators, improve metabolic parameters in insulin-dependent animal models. In some cases, they have fewer side effects than thiazolidinediones or fenofibrate as complete agonists [12,13].

In the present work, it was considered that the modulation of the nuclear factor kappa-light-chain-enhancer of activated B cells (NF-κB) expression might be related to the anti-inflammatory effect of the PPAR-γ ligand. PPAR-γ is a nuclear receptor and can inhibit the DNA-binding activity of NF-κB [14]. The transcription factor NF-κB plays a critical role in controlling inflammation and cell death (apoptosis). Apoptosis, inflammation, and cytokine production are regulated in part by PPAR-γ. Research suggests that the expression and activation of PPAR-γ could suppress isoproterenol (ISO)-induced inflammation [15,16]. Therefore, the modulation of PPAR-γ and NF-kB signaling pathways may be a potential method for treating MI in diabetic patients. In addition, the anti-inflammatory impact of MI is one of the primary factors contributing to its effectiveness [17]. The activation of an intracellular signaling cascade is initiated at MI by the phosphorylation of NF-κB. Finally, it causes various pathological and physiological changes by inducing proinflammatory cytokines such as tumor necrosis factor α (TNF-α), interleukin 6 (IL-6), interleukin 1β (IL-1β), and other inflammation-related proteins. These cytokines are responsible for a variety of changes [18]. 

**Scheme 1:**
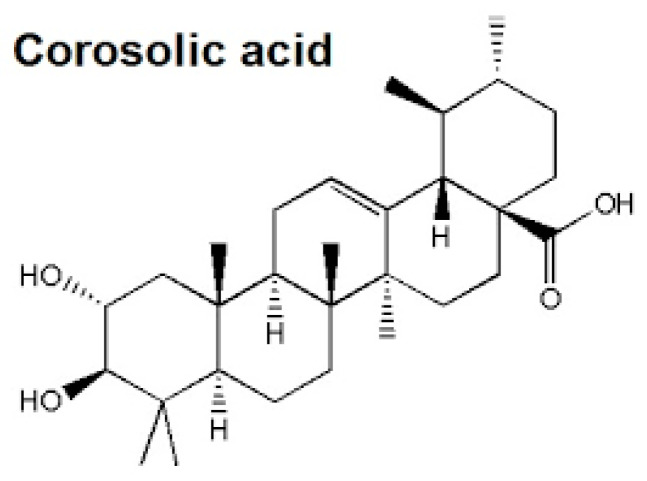
Chemical structure of corosolic acid.

Several different plant species, including *Vaccinium macrocarpon*, *Ugni molinae*, *Eriobotrya japonica*, *Perilla frutescens*, *Glechoma longituba*, *Potentilla chinensis*, *Rubus biflorus*, *Phlomis umbrosa*, etc., have been tested and shown to contain the phytoconstituent known as corosolic acid. [19]. Corosolic acid (Scheme 1) is also known as 2α-hydroxyursolic acid, with the molecular formula C_30_H_48_O_4_ and a molecular weight of 472.70 g/mol. The pentacyclic triterpenoid corosolic acid, which is relatively abundant, has attracted considerable interest in recent years because of its potential to control diabetes. It is also known as “phyto-insulin” or “plant insulin” because of its excellent antidiabetic effects [20]. Various pharmacological studies have shown that corosolic acid has anti-obesity [21], anti-inflammatory [22], anti-hyperlipidaemic [22], anti-viral, and anti-cancer properties [23]. Based on these considerations, the present study aimed to determine whether corosolic acid has a cardioprotective effect in diabetic rats and to explore the mechanisms involved. Specifically, the researchers were interested in reconnoitering the effects of corosolic acid on the heart.

## 2. Results

### 2.1. Effect of Corosolic Acid on Body Weights and Heart Weights

**Figure 1:**
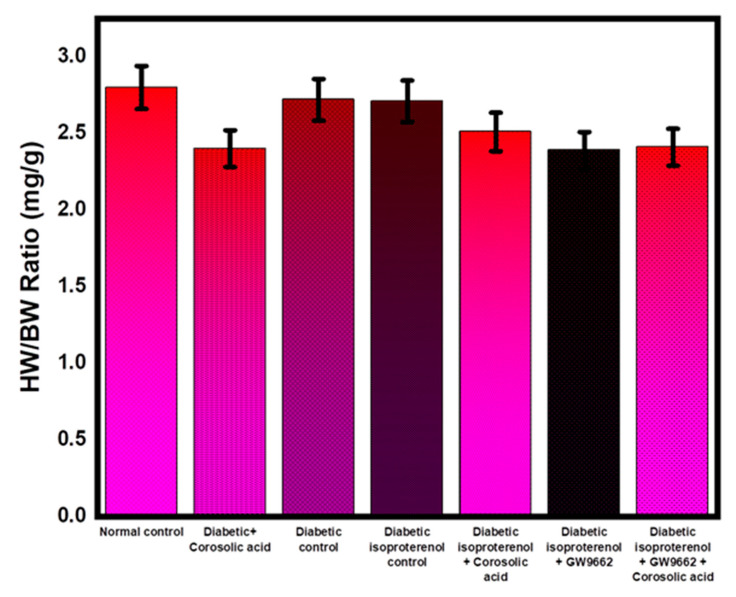
The effect of corosolic acid on the ratio between animal heart weight and body weight. The data are expressed as mean ± SEM (*n* = 6).

Despite the various treatment regimens, no statistically significant differences were seen in the rat’s body weight, heart weight, or ratios of body weight to heart weight (Figure 1). Table 1 presents the findings of the study. 

### 2.2. Effect of Corosolic Acid on Blood Glucose Level

**Figure 2:**
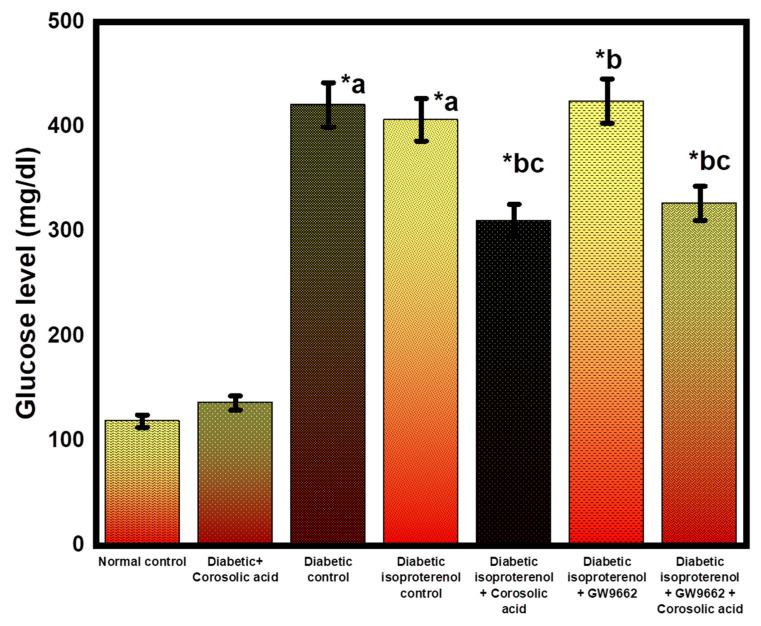
Effects of corosolic acid on glucose level in different groups. The data are expressed as mean ± SEM (*n* = 6). * *p* ≤ 0.05 is considered significant. ^a^ normal control vs. diabetic control and diabetic isoproterenol control; ^b^ diabetic control vs. corosolic acid and GW9662 treatments; and ^c^ diabetic isoproterenol control vs. corosolic acid and GW9662 treatments.

Diabetic rats given corosolic acid had significantly lower blood glucose levels than the diabetic control and diabetic ISO control groups (Figure 2).

### 2.3. Effect of Corosolic Acid on the Hemodynamic Parameters and Heart Rate

**Figure 3:**
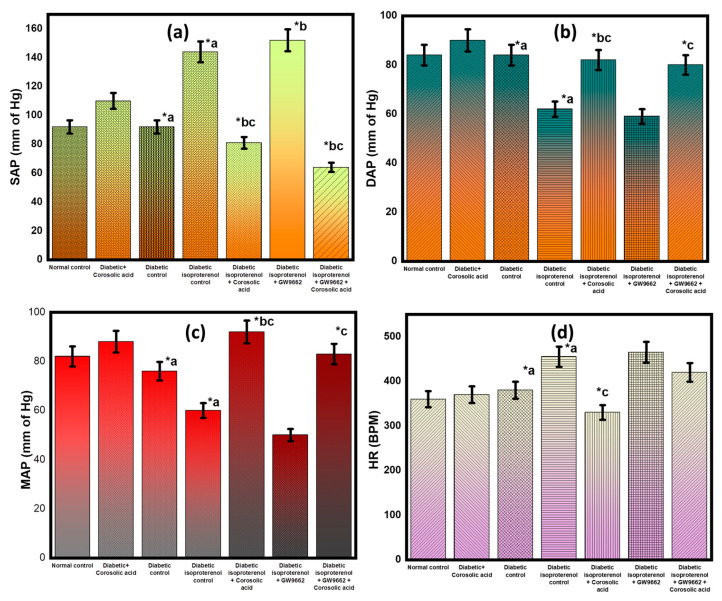
Effects of corosolic acid on heart rate and hemodynamic parameters in different groups: (**a**) SAP; (**b**) DAP; (**c**) MAP; (**d**) HR. The data are expressed in the form of mean ± standard error average (SEM). The significance was determined by one-way ANOVA followed by the Bonferroni’s post hoc test: * *p* ≤ 0.05 is considered significant. ^a^ normal control vs. diabetic control and diabetic isoproterenol control; ^b^ diabetic control vs. corosolic acid and GW9662 treatments; and ^c^ diabetic isoproterenol control vs. corosolic acid and GW9662 treatments. (SAP: systolic arterial pressure; DAP: diastolic arterial pressure; MAP: mean arterial pressure; HR: heart rate).

The values of systolic arterial pressure (SAP), diastolic arterial pressure (DAP), and mean arterial pressure (MAP) were significantly lower in the diabetic isoproterenol rats than in the diabetic control group (*p* ≤ 0.05). All these hemodynamic changes in diabetic rats were consistent with the ischemic cardiac damage caused by isoproterenol. Compared with the diabetic isoproterenol-treated group, the corosolic acid-treated group showed improved hemodynamic parameters (Figure 3). Hemodynamic parameters were not significantly improved by GW9662 but instead worsened. There was no statistical evidence for the efficacy of GW9662. Combined with corosolic acid, GW9662 even worsened blood pressure and heart rate.

### 2.4. Effect of Corosolic Acid on Cardiac Injury Markers

**Figure 4:**
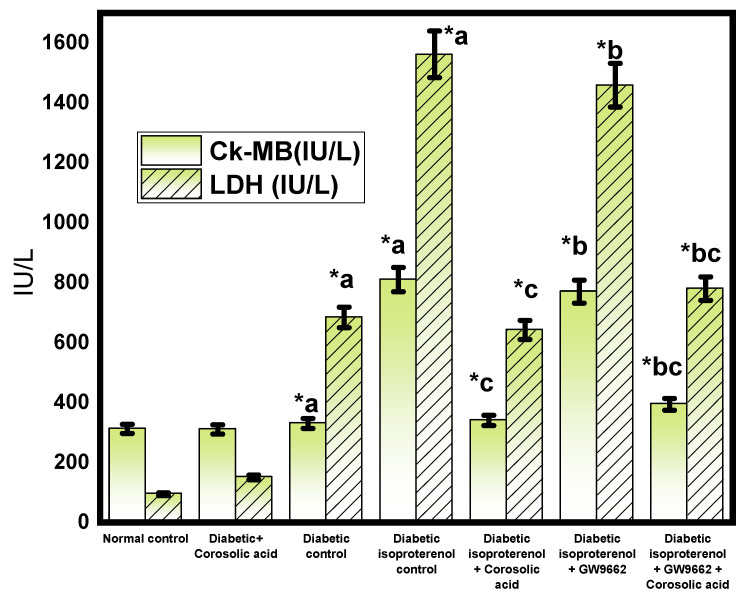
The effect of corosolic acid on the markers of heart disease in different groups. The data are expressed as mean ± SEM. * *p* ≤ 0.05 is considered significant. ^a^ normal control vs. diabetic control and diabetic isoproterenol control; ^b^ diabetic control vs. corosolic acid and GW9662 treatments; and ^c^ diabetic isoproterenol control vs. corosolic acid and GW9662 treatments. (IU: international unit; CK-MB: creatine kinase on myocardial bundle; LDH: lactate dehydrogenase).

The biochemical markers creatinine kinase at the myocardial bundle (CK-MB) and lactate dehydrogenase (LDH) were used to evaluate the effects of different therapies on cardiac membrane integrity. This was done to examine the effects of each measurement parameter side by side and to compare them. As might be expected, the treatment with isoproterenol significantly decreased the values of all these indicators in diabetic rats. The administration of corosolic acid resulted in a statistically steep increase in the myocardial levels of both CK-MB and LDH. This proves that the integrity of the cardiac membrane was not affected. The protective effect of corosolic acid was attenuated by pretreatment with GW9662 (Figure 4). Myocardial damage was extreme in the GW9662 group, as shown by the increased levels of markers of cardiac damage.

### 2.5. Effect of Corosolic Acid on Antioxidant Parameters

**Figure 5:**
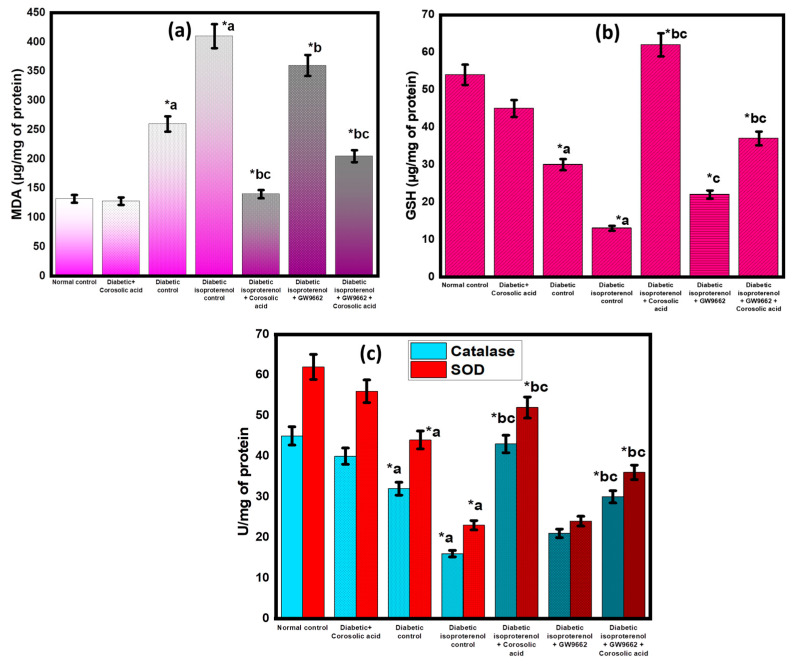
The effect of corosolic acid on oxidative stress in different groups: (**a**) MDA content; (**b**) GSH; (**c**) catalase and SOD. The data are expressed as mean ± SEM. * *p* ≤ 0.05 is considered significant. ^a^ normal control vs. diabetic control and diabetic isoproterenol control; ^b^ diabetic control vs. corosolic acid and GW9662 treatments; and ^c^ diabetic isoproterenol control vs. corosolic acid and GW9662 treatments.

In rats administered diabetic isoproterenol or diabetic isoproterenol with GW9662 and corosolic acid, there was a significant increase (*p* ≤ 0.05) in chemicals interacting with thiobarbituric acid, which may indicate oxidative damage to the cell membrane. Lower levels of glutathione (GSH), superoxide dismutase (SOD), and catalase were detected in the myocardial tissue (*p ≤* 0.05). Corosolic acid therapy significantly increased endogenous antioxidant levels and decreased lipid peroxidation compared to the diabetic control group and the diabetic isoproterenol group. Based on the data from the hemodynamic measurements, it can be concluded that treatment with GW9662 decreased the antioxidant effect of corosolic acid (Figure 5).

### 2.6. Effect of Corosolic Acid on PPAR-γ Expression in Different Groups

**Figure 6:**
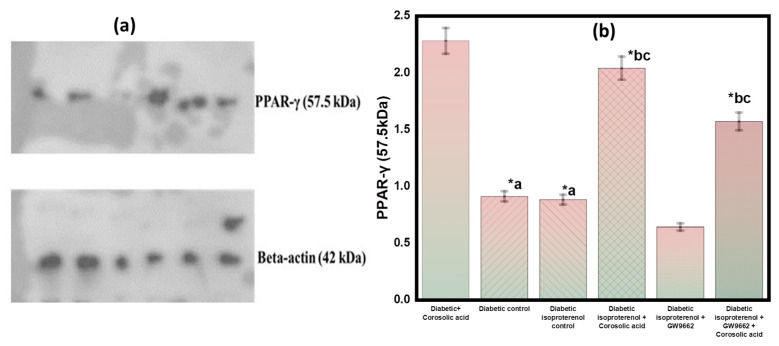
(**a**) PPAR-γ expression. (**b**) The effects of corosolic acid on the expression of PPAR-γ in different groups were quantified by Western blot analysis. The data are expressed as mean ± SEM. * *p* ≤ 0.05 is considered significant. ^a^ normal control vs. diabetic control and diabetic isoproterenol control; ^b^ diabetic control vs. corosolic acid and GW9662 treatments; and ^c^ diabetic isoproterenol control vs. corosolic acid and GW9662 treatments.

The expression of PPAR-γ in the cardiac tissue, the glucose control group, and the isoproterenol control groups was significantly lower. There was a significantly increased PPAR-γ protein expression statistically (*p* ≤ 0.05). The expression of PPAR-γ in diabetic isoproterenol rats and diabetic isoproterenol rats plus GW9662 rats was comparable. The PPAR-γ expression was increased when corosolic acid was coupled with GW9662 compared with GW9662 alone. These results indicate that PPAR-γ expression is critical for controlling the diabetic benefit of corosolic acid (Figure 6).

### 2.7. Effect of Corosolic Acid on Inflammatory Cytokines

**Figure 7:**
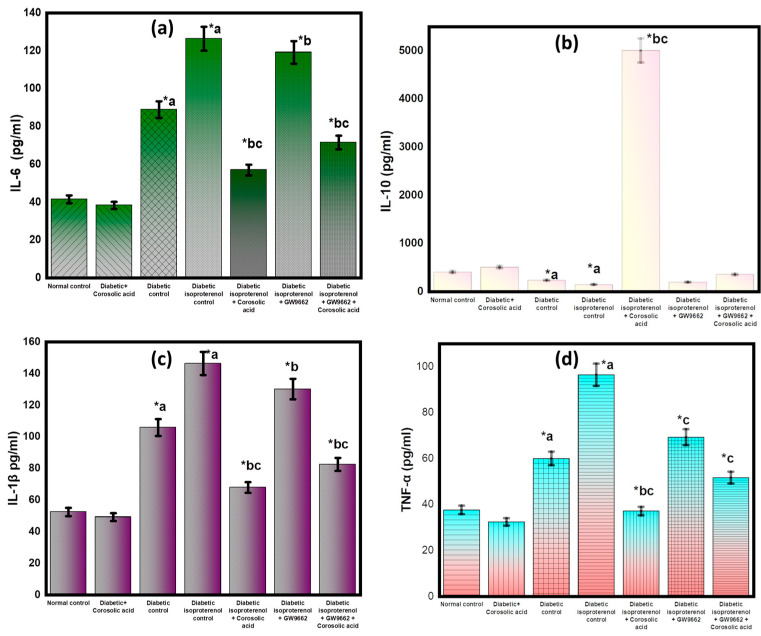
The effects of corosolic acid on proinflammatory cytokines (**a**) IL-6; (**b**) IL-10; (**c**) IL-1β; (**d**) TNF-α. The data are expressed as mean ± SEM. * *p* ≤ 0.05 is considered significant. ^a^ normal control vs. diabetic control and diabetic isoproterenol control; ^b^ diabetic control vs. corosolic acid and GW9662 treatments; and ^c^ diabetic isoproterenol control vs. corosolic acid and GW9662 treatments.

The levels of proinflammatory cytokines (TNF-α, IL-1, IL-6, and IL-10) and anti-inflammatory cytokines (IL-10) in rats with MI are compared to those in the normal control group in Figure 7. After the development of MI, there was a significant increase in the expression of these proinflammatory cytokines. The levels of corosolic acid in the myocardium were significantly lower after treatment, whereas the levels of the anti-inflammatory IL-10 were significantly higher. The data analysis is shown in Figure 7.

### 2.8. Histopathological Analysis

The histopathological results differed significantly between the control and experimental groups. Compared with the diabetic control groups and the diabetic isoproterenol control groups, the ISO + corosolic acid group had less histological damage to the heart (Table 2). This indicates that corosolic acid protects against myocardial infarction after isoproterenol has already caused it.

## 3. Discussion

The oxidative stress, inflammation, and apoptosis associated with diabetic cardiomyopathy can be replicated in animal studies [8]. ISO was used to mimic acute myocardial infarction induced by diabetes in rats using the diabetes model generated in rats by STZ [10]. ISO is a non-selective β-adrenergic agonist that induces MI in rats by continuously activating the adrenergic receptor. It accelerates the heartbeat, makes the myocardium work harder, and causes dilation of the mesenteric and renal vessels. As a result, there is an increase in lipid peroxidation, free radical formation, and depletion of the antioxidant defense system. All these physiological and biochemical changes induced by ISO contribute to inflammation, apoptosis, and necrotic changes in the myocardium and the development of MI. ISO-induced MI in rats is consistent with the pathogenic and marked hemodynamic and tissue changes reported in humans’ MI [11]. The ability of ISO to produce acute MI in rats with STZ-induced diabetes is a novel paradigm that has the potential to be used in studies to evaluate the efficacy of therapy for acute MI associated with acute diabetes and the molecular processes behind such treatment.

STZ is known to be a potent cytotoxic agent for rat pancreatic beta cells. Briefly, STZ causes DNA damage to insulin-secreting cells, leading to DNA repair mechanisms, mitochondrial dysfunction, and ATP loss. It reduces ATP formation by interfering with mitochondrial respiratory complexes. STZ causes hyperglycemia in rats and inhibits aconitase activity [24]. Similar to STZ, isoproterenol increases free radical production in the body, impairing the body’s antioxidant defenses. Cardiac dysfunction and associated histological damage are the results of calcium excess. The myocardial structural changes caused by isoproterenol are similar to those seen in patients after MI [25].

Myocardial damage due to oxidative stress is prevented by endogenous antioxidants such as superoxide dismutase (SOD), catalase enzymes, and glutathione (GSH) [26]. We observed that the administration of ISO and GW9662 to diabetic rats significantly decreased SOD/catalase enzyme activity and GSH levels in the cardiac tract. These results suggested an increase in oxidative stress. Treatment with corosolic acid resulted in a significant improvement in antioxidant enzyme activity and an increase in GSH levels. In a rat model of MI, corosolic acid is also known to decrease reactive oxygen species (ROS) formation and prevent oxidative damage to the myocardial system [27]. Our findings support the notion that corosolic acid may have antioxidant properties through the PPAR-γ pathway.

Research has shown that lipid peroxidation has the most significant and most damaging impact on MI-related cellular damage [28]. ISO and GW9662, both of which increase oxidative stress, are responsible for the significant increase in malondialdehyde (MDA) levels observed in the hearts of experimental participants. ISO may have led to an accumulation of lipids in the hearts of rats, resulting in persistent damage to the myocardial membrane, which in turn led to increased MDA concentrations [29]. In addition, GW9662 was shown to decrease the amount of lipid peroxidation in the heart of diabetic rats with myocardial infarction [30]. The nuclear receptor known as PPAR-γ is an important player in the process of adipocyte differentiation, maintenance of glucose homeostasis, and control of inflammation [31]. The PPAR-γ signaling pathway controls several of the biological activities that occur in the cardiovascular system. This nuclear superfamily of transcription factors is responsible for inhibiting the development of inflammatory cytokines. It does so by modulating the activity of other transcription factors that are related to each other [31]. PPAR-γ agonists reduce the extent and the severity of infarction in a myocardial infarction model with ischemia. However, the efficacy of PPAR-γ agonists varies widely in terms of their ability to protect against MI. According to research published in [32], corosolic acid, a potent PPAR-γ agonist, decreased PPAR-γ levels by acting on central hypoxia-induced factor alpha. GW9662, a selective irreversible PPAR-γ antagonist, was shown to decrease PPAR-γ expressions in cardiac tissue [33]. On the other hand, the effect of corosolic acid was improved in PPAR-γ expressions. Thus, these results strengthen the evidence that the PPAR-γ pathway is involved in the effects of corosolic acid [34,35].

The results of the current study showed that the cardioprotective benefits of corosolic acid include the restoration of cardiovascular function, preservation of endogenous antioxidants, the histological rescue of myofibrils, and the reduction of lipid peroxidation in cardiac tissue [36,37]. In contrast, the expression of PPAR-γ was enhanced by the presence of corosolic acid. The discovery that concomitant therapy with the PPAR-γ antagonist GW9662 significantly attenuates the cardiovascular protection afforded by corosolic acid supports this theory. This work conclusively demonstrates that the activation of the PPAR-γ pathway is associated with the cardioprotective effect of corosolic acid [38,39].

Lower systolic, diastolic, and mean arterial blood pressure values were shown in this study to indicate diabetes- and MI-related hemodynamic impairments. On the other hand, these are signs that myocardial activity is decreasing. In contrast, the PPAR-γ antagonist GW9662 worsened blood pressure and heart rate [40]. Finally, the cardioprotective effect of corosolic acid was abolished by the combined treatment with GW9662. The activation of the PPAR-γ pathway may be responsible, at least in part, for the observed reversal of the cardioprotective effect of GW9662-corosolic acid. Modifying PPAR-γ has therapeutic benefits in treating cardiac injury [41]. However, GW9662 treatment exacerbated the damage in diabetic mice MI. It is well known that PPAR-γ plays a crucial role in controlling apoptosis [36]. Therefore, the fact that GW9662, a PPAR-γ antagonist, abolished the cardiovascular protective effect of corosolic acid highlights this receptor’s role in the beneficial effects of corosolic acid on the cardiovascular system.

The integrity of the myocardial membrane is compromised during an MI, resulting in the release of components of the isoenzymes CK-MB and LDH from the myocardial membrane into the blood [39]. A decrease in the activity of these enzymes was observed in the myocardium of diabetic rats treated with isoproterenol. Previous studies [29,30] have shown that this effect follows the same results. After treatment with corosolic acid, the activity of these enzymes normalized, and the cell membrane of cardiomyocytes was shown to become more stable. In the cardiac tract, the activity of these enzymes was decreased after GW9662 had been administered previously. This discovery could be considered further evidence of the function of PPAR-γ in the protective effect of corosolic acid on the myocardium.

The efficacy of corosolic acid in diabetic rats was also confirmed and supported by histological studies. Cardiac damage and inflammation were observed in rats administered STZ plus isoproterenol. In diabetic rats with MI treated with corosolic acid, these histological abnormalities in the myocardium were avoided. According to the results of our study, GW9662 was able to counteract the therapeutic effects of corosolic acid in treating myonecrosis, inflammation, and edema.

## 4. Materials and Methods

All experimental procedures were performed after approval by the Standing Committee on Bioethical Research (SCBR), College of Pharmacy, Prince Sattam Bin Abdulaziz College, Saudi Arabia (research approval number SCBR-037-2022). For the purpose of the study, adult male Wistar rats weighing between 200 and 250 g were maintained in a standard laboratory environment and had access to food and water at all times.

### 4.1. Chemicals

Corosolic acid, isoproterenol, streptozotocin, and GW9662 were purchased from Sigma-Aldrich, Bangalore, India. GSH, SOD, catalase, and 1,1,3,3-tetraethoxypropane assay kits were used from Sigma-Aldrich, Bangalore, India. Quantitative detection kits for CK-MB isoenzyme, LDH (Span diagnostics, Hyderabad, India), and PPAR-γ antibodies (sc-271392) (Santa Cruz Biotechnology, Inc., Santa Cruz, CA, USA) were used.

### 4.2. Experimental Study

#### 4.2.1. Induction of Diabetes

Rats received intraperitoneal STZ injections at a dose of 55 mg/kg, resulting in the development of diabetes. Prior to administration, the STZ solution was prepared in a 0.1 M glacial citrate buffer (pH 4.5). On the third day after STZ injection, the rats whose blood glucose levels were above 250 mg/dL were selected as diabetic rats for further study [24]. We randomly assigned diabetic rats to different groups and six rats in each group. Table 3 shows the groups and treatments.

#### 4.2.2. Estimation of Food and Water Consumption

We monitored the body weight, water intake, and food intake of rats throughout the study period.

#### 4.2.3. Estimation of Heart Rate and Blood Pressure

A rat tail cuff plethysmograph and a pressure gauge were used to measure hemodynamic parameters, heart rate, and systolic blood pressure (SBP) at day 28 in the group [40]. Hemodynamic parameters were measured, including the mean arterial pressure (MAP), the systolic arterial pressure (SAP), and the diastolic arterial pressure (DAP).

#### 4.2.4. Estimation of the Cardiac Injury Markers and Oxidative Stress

In an ice-cold phosphate buffer (50 mM, pH 7.4) (Sisco Research Laboratories Pvt. Ltd. (SRL)—Mumbai, India), 10% homogeneous heart tissue homogenates were prepared from each rat. Assays of lipid peroxidation, GSH content, catalase activity, CK-MB, and LDH were performed on aliquots of the supernatant after centrifuging at 2000 rpm for 20 min at 4 °C, followed by centrifugation at 3000 rpm for 20 min [27].

#### 4.2.5. Estimation of Lipid Peroxidation (MDA Content)

MDA content was used to detect lipid peroxidation in the cardiac tissue, as reported by Buwa et al. (2016) [42]. An acetic acid solution (Sisco Research Laboratories Pvt. Ltd. (SRL)—Mumbai, India) of pH 3.5 was mixed with 1.5 mL of thiobarbituric acid (0.8%) (HiMedia Laboratories Pvt. Ltd., Maharashtra, India) and 1.5 mL of sodium dodecyl sulfate, in equal amounts, with 0.2 mL of tissue homogenates. After heating to 95 °C for 60 min, the reaction mixture was cooled on ice. The mixture was centrifuged at 5000 rpm for 20 min using a tabletop centrifuge (Sigma 3-30, Sigma, Steinheim, Germany) for 20 min after being cooled in 1 L of distilled water and 5 mL of n-butanol:pyridine solution (15:1 *V*/*V*). At 532 nm, the organic layer was separated, and the absorbance rate measured. The MDA content was given in terms of µg/mg of protein.

#### 4.2.6. Estimation of Glutathione Content

A method described by Salbitani et al. (2017) was used to estimate the GSH content [43]. We mixed 100 µL of tissue homogenate with 10% trichloroacetic acid (Sigma-Aldrich, Bangalore, India) and poured it into a test tube. Then, 5′-dithiobis (2-nitrobenzoic acid) (Sigma-Aldrich, Bangalore, India) and 3.0 mL of phosphate buffer (pH 8.4) were added to the supernatant after centrifugation at 5000 rpm for 10 min. A spectroscopy measurement at 412 nm was performed within 10 min of mixing the mixture. Protein concentrations of GSH were expressed as µg/mg.

#### 4.2.7. Estimation of Catalase Activity

The catalase activity was determined using the method described by Sah and Nagarathana (2016) [44]. To 50 µL of tissue supernatants, we added 50 mM phosphate buffer (pH 7) and 0.1 mL 30 mM hydrogen peroxide. At 240 nm, the optical density of the sample was measured at every 5 s for 30 s. The catalase activity was expressed in U/mg of protein.

#### 4.2.8. Estimation of Superoxide Dismutase Activity

According to Senthilkumar et al. (2021), the MDA content was used to detect lipid peroxidation in cardiac tissue [45]. In total, 1 mM ethylenediaminetetraacetic acid (EDTA) (Sigma-Aldrich, Bangalore, India) solution and 500 mM Na_2_CO_3_ (Sigma-Aldrich, Bangalore, India) were added to 25 µL of tissue supernatant, along with 100 µL of 240 µM/nitroblue tetrazolium (NBT; Sigma-Aldrich, St. Louis, MO, USA), 640 l µL of distilled water, and 25 µL of 10 mM hydroxylamine. Spectrophotometric measurements at 560 nm were carried out at intervals of one to three minutes. Enzyme activity was expressed in the form of U/mg proteins.

#### 4.2.9. Western Blot Analysis for PPAR-γ

The cardiac muscle tissue was homogenized in the lysis buffer of the radioimmunoprecipitation assay (RIPA), and the protein content was calculated. A Western blot analysis was performed as in a previously described method [46]. The sodium dodecyl sulfate-polyacrylamide gel (SDS-PAGE) method was used to separate myocardial tissue. Then, 5% bovine serum albumin was applied to a nitrogen cellulose membrane for 2 h to block it and transfer it to another nitrogen cellulose membrane. It was also incubated with an original rat antibody (PPAR-γ) for 12 h at 4 °C. Densitometric scanning was conducted on the obtained lines in order to quantify the level of PPAR-γ expression.

#### 4.2.10. Measurement of Inflammatory Cytokines by Enzyme-Linked Immunosorbent Assay (ELISA)

A homogenizer was used to homogenize the left ventricles from mice, and the cells were centrifuged at 5000 rpm for 15 min at 4 °C for 15 min. The levels of IL-6, IL-1, and TNF-α in the supernatant were determined using an ELISA kit and analyzed in accordance with the guidelines provided by the manufacturer. In brief, anti-mouse antibodies were coated onto the ELISA plate [47]. For each well, 100 µL of the test sample or standard was added and incubated for 2 h at 37 °C. Each well was incubated for one hour at 37 °C with biotinyl anti-mouse antibody solution. The incubation was followed by three washes of the liquid. Incubation at 37 °C for one hour was followed by the addition of secondary antibodies stabilized with horseradish peroxidase to each well. An incubation at room temperature was followed by washing the plate with a 3,3′,5,5′-Tetramethylbenzidine chromogen solution. Using a microplate reader, we measured the absorbance rate at 450 nm after adding a stop solution to each well. Based on a standard curve, cytokine concentrations were determined [48].

#### 4.2.11. Histopathological Analysis

After the study was completed, histopathological examinations were conducted. The histopathological analysis was based on the following classifications (damage and changes in the myocardium): (a) if no change or appeared to be normal, it was counted as grade 0; (b) having mild or focal myocyte damage or small multifocal degeneration with a mild inflammatory process was counted as grade 1; (c) moderate myocyte damage or myofibrillar degeneration with diffuse inflammatory process was counted as grade 2; (d) severe myocyte damage or necrosis with a diffuse inflammatory process was counted as grade 3 [49,50].

#### 4.2.12. Statistical Analysis

Data are presented as mean ± standard error mean (SEM). Graph Pad Prism software, version 7.04 (GraphPad Software, Boston, MA, USA, 02110), was used to test statistical significance, using a one-way analysis (ANOVA) followed by a Bonferroni post hoc test. A *p* < value of 0.05 was considered statistically significant.

## 5. Conclusions

Diabetes increases the risk of cardiovascular disease and mortality by two to four times compared to non-diabetics [51]. Diabetics are at a greater risk of myocardial infarction due to the presence of a number of risk factors, including hyperglycemia, dyslipidemia, oxidative stress, and inflammation. PPAR-γ is a master regulator of glucose, fatty acid, and lipoprotein metabolism and also influences inflammation, cell proliferation, and apoptosis [52]. Corosolic acid, a component of banaba leaves, can activate PPAR-γ. By using corosolic acid, hemodynamic disturbances could be prevented, left ventricle function could be restored, and redox balance could be maintained. Rats are protected from MI by attenuating myonecrosis, swelling, and cell death. The increased expression of PPAR-γ in the myocardium of rats receiving corosolic acid highlights the role of PPAR-γ pathway activation in the cardioprotective effects of corosolic acid.

## 6. Future Perspectives

The pharmacological activation of PPAR-γ improves several metabolic parameters in humans. However, there are no marketed drugs that target PPAR-γ. Further exploration of the mode of action of corosolic acid may lead to the development of a novel drug that can be employed either individually or in combination with other drugs in the therapeutic management of diabetes and associated cardiovascular complications. In addition, extensive studies are underway in various pharmaceutical companies and academic institutions to develop PPAR-γ agonists with multiple or partial receptor activity in an effort to improve treatment strategies in the management of diabetes or metabolic syndromes and associated cardiovascular complications.

## Figures and Tables

**Table 1:** Effect of corosolic acid on weights in different experimental groups.

Groups	Heart Weight (g)	Body Weight (g)
Normal control	0.65 ± 0.04	243.4 ± 3.23
Diabetic + Corosolic acid	0.60 ± 0.05	238.4 ± 4.50
Diabetic control	0.62 ± 0.03	235 ± 3.61
Diabetic isoproterenol control	0.59 ± 0.08	217 ± 3.32
Diabetic isoproterenol + Corosolic acid	0.61 ± 0.04	239 ± 2.82
Diabetic isoproterenol + GW9662	0.56 ± 0.07	209 ± 3.41
Diabetic isoproterenol + GW9662 + Corosolic acid	0.55 ± 0.04	228 ± 4.21

**Table 2:** The effect of corosolic acid on histopathological grades of myocardial injury in rats.

Groups	Grade 0	Grade 1	Grade 2	Grade 3
Normal control	0	1	0	0
Diabetic + Corosolic acid	0	0	0	0
Diabetic control	3	2	2	1
Diabetic isoproterenol control	0	2	2	3
Diabetic isoproterenol + Corosolic acid	0	1	1	1
Diabetic isoproterenol + GW9662	0	2	2	2
Diabetic isoproterenol + GW9662 + Corosolic acid	1	2	2	1

**Table 3:** Grouping of animals.

Groups	Subjects	Treatment Given
Group I	Normal control	Vehicle (distilled water) for 28 days.
Group II	Diabetic rats treated with corosolic acid 50 mg/kg	50 mg/kg/day of corosolic acid for 28 days by oral route in diabetic rats.
Group III	Diabetic control	28 days of oral administration with distilled water as carrier (1 mL/kg). On days 26 and 27, rats received 0.2 mL saline subcutaneously as vehicle control.
Group IV	Diabetic isoproterenol-treated	The above vehicle was administered to the rats in this group for 28 days, and isoproterenol (100 mg/kg) was also administered subcutaneously to the rats on days 26 and 27.
Group V	Diabetic isoproterenol-treated rats administered with corosolic acid 50 mg/kg	Rats received 50 mg/kg/day of corosolic acid orally for 28 days. On days 26 and 27, rats were injected subcutaneously with isoproterenol (100 mg/kg).
Group VI	Diabetic isoproterenol-treated rats administered with GW9662	Rats in this group received GW9668 1 mg/kg/day by intraperitoneal (i.p.) injection on day 28, and 100 mg/kg isoproterenol by subcutaneous injection on days 26 and 27.
Group VII	Diabetic isoproterenol-treated rats administered with corosolic acid and GW9662	This group of rats received oral corosolic acid (50 mg/kg) every day. On 28th day, corosolic acid was given just 15 min before GW9662 administration. On the last two days of therapy, 100 mg/kg isoproterenol was administered subcutaneously to the rats in this group [29,41].

## Data Availability

The data presented in this study are available on request from the corresponding author.

## Sample Availability

Sample of the Corosolic acid is available from the authors.
